# Connecting PM_2.5_ Exposure to Insulin Resistance: Oxidative Stress May Be an Intermediate Step

**DOI:** 10.1289/ehp.124-A236

**Published:** 2016-12-01

**Authors:** Julia R. Barrett

**Affiliations:** Julia R. Barrett, MS, ELS, a Madison, WI–based science writer and editor, is a member of the National Association of Science Writers and the Board of Editors in the Life Sciences.

Animal studies and human epidemiological research suggest that exposure to fine particulate matter (PM_2.5_) increases the risk of developing type 2 diabetes. But it is unclear how PM_2.5_ exposure might lead to systemic insulin resistance, the cause of type 2 diabetes. The authors of a new study in mice conclude that oxidative stress in the lungs may be an intermediate step between exposure to PM_2.5_ and vascular insulin resistance, a precursor of systemic insulin resistance.^1^

Type 2 diabetes arises from the body’s inability to respond to insulin produced by the pancreas. This results in high blood glucose levels and potentially fatal health complications. The worldwide prevalence of adult diabetes (both type 1 and type 2) doubled from 4.7% in 1980 to 8.5% in 2014, when the global prevalence reached approximately 422 million individuals.^2^ An increase in type 2 diabetes cases has also been observed among children and teens, a population that was rarely affected 20 years ago.^3^

**Figure:**
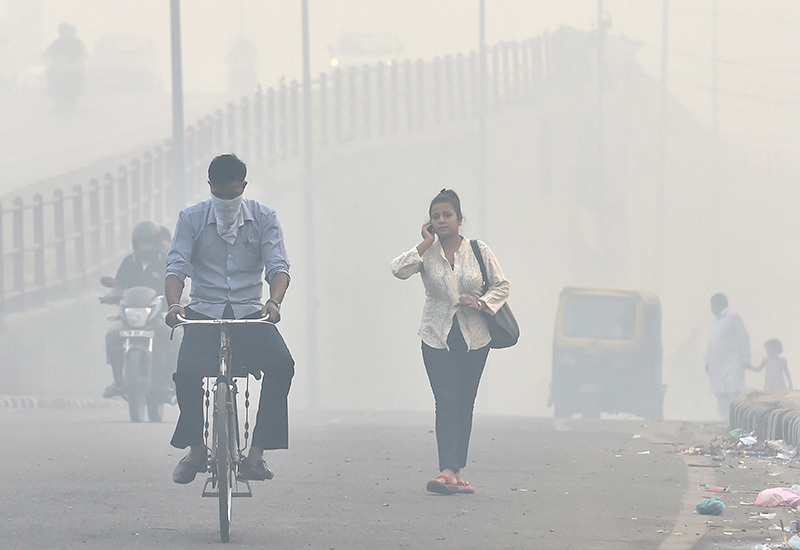
The authors of a new mouse study hypothesize that vascular insulin resistance may be caused when PM_2.5_ exposure triggers oxidative stress in the lungs. The experimental study doesn’t replace the need for human research but it could offer insight into how air pollution might increase the risk of insulin resistance and type 2 diabetes. © Hindustan Times/Getty Images

Much of the increase among adults has been attributed to the rising incidence of obesity and lack of exercise, which are well-established risk factors for type 2 diabetes.^1^ ^,^ ^2^ But exposure to air pollutants, including PM_2.5_, also may play a role.^1^ ^,^ ^4^ ^,^ ^5^

“We have a lot of experimental studies as well as epidemiological studies that show that air pollution at low levels [may cause] insulin resistance and susceptibility to type 2 diabetes,” says Sanjay Rajagopalan, an adjunct professor at the University of Maryland School of Medicine, who was not involved in the new animal study. “It’s not a strong signal like consuming a high-fat diet or lacking exercise—those are believed to be the dominant factors—but don’t underestimate the importance of air pollution,” says Rajagopalan.

Previous experimental research in mice showed that vascular insulin resistance (which occurs in blood vessels) develops prior to systemic resistance (which occurs in muscle, liver, and adipose tissue).^6^ Both systemic and vascular insulin resistance have been associated with PM_2.5_ exposure in the context of a high-fat diet and obesity in animals.^7^

Oxidative stress is widely thought to be an intermediate step between PM_2.5_ exposure and toxic effects. The authors of the new study hypothesized that vascular insulin resistance may be caused when PM_2.5_ exposure triggers oxidative stress in the lungs. They tested this hypothesis in a series of experiments in which they fed mice a control diet or a high-fat diet and exposed them to either filtered air or concentrated PM_2.5_ (CAP) for 9 or 30 days.

CAP exposure was associated with vascular insulin resistance in the mice, in addition to vascular inflammation and dysfunction, even in mice on the control diet. The authors noted that CAP exposure was associated with an increase in plasma markers of oxidative stress and lipid peroxidation, and that pretreating mice with an antioxidant prevented some of the early CAP-related changes in vascular insulin signaling. They also found that CAP exposure led to increased expression of genes in the lungs that are specifically induced by oxidative stress. Mice that were bred to overexpress one of these genes only in the lungs had less of a response to CAP.

The new findings could explain how air pollution may contribute not only to metabolic disease but also cardiovascular disease, another condition linked with PM_2.5_ exposure. In addition, the model suggests that not only obesity but also any other condition involving oxidative stress may increase the susceptibility to harm from PM_2.5_. “The most important next step is to figure out how the oxidative stress in the lungs gets to the vasculature,” says lead author Petra Haberzettl, an assistant professor at the University of Louisville School of Medicine. “Everybody could be affected by air pollution exposure,” Haberzettl says, “but someone with asthma, for example, who is lean and has no metabolic problems, could be more [susceptible] to PM exposure.”

Rajagopalan cautions that this experimental study does not replace the need for human studies, nor does it completely replicate the human experience. “Nonetheless,” he says, “the findings provide insight into how air pollution, through lung-mediated mechanisms, might alter susceptibility to what’s considered a systemic problem—insulin resistance and type 2 diabetes.”
